# Mazri (*Nannorrhops ritchiana* (Griff) Aitch.): a remarkable source of manufacturing traditional handicrafts, goods and utensils in Pakistan

**DOI:** 10.1186/s13002-020-00394-0

**Published:** 2020-08-17

**Authors:** Abdullah, Shujaul Mulk Khan, Andrea Pieroni, Zahoor ul Haq, Zeeshan Ahmad

**Affiliations:** 1 Department of Plant Sciences, Quaid-i-Azam University, Islamabad, Pakistan; 2 University of Gastronomic Sciences, Pollenzo/Bra, Italy; 3 Department of Medical Analysis, Tishk International University, Erbil, Kurdistan Italy

**Keywords:** Mazri palm, Ethnobotany, Handicrafts, Pakistan, Biological conservation

## Abstract

**Background:**

Mazri palm (*Nannorrhops ritchiana* (Griff) Aitch.) is a member of the family Arecaceae, native to Pakistan, Iran, Afghanistan, Oman, and Saudi Arabia. In Pakistan, it is used since long time for various purposes. This species plays a significant cultural and economic role in the daily lives of many rural areas in Pakistan and adjacent countries. However, the handcrafted products made up of this palm are often mainly known by specific local communities rather than by a broader range of people.

**Methods:**

Eighty-six structured and semi-structured interviews were conducted from Mazri growing areas, villages, and markets of urban centers during the fieldwork that was conducted in diverse regions of Pakistan. Interviewees included 27 Mazri farmers, 17 locals retaining Traditional Knowledge in handcrafting Mazri palm (12 were men and 5 were women), 23 handicrafts experts (21 were men and 2 were women), and 19 sellers. The age of the informants ranged from 14 to 83 years. Study participants shared detailed information about various traditional utilizations of the Mazri palm.

**Results:**

Mature leaves of Mazri palm are used to produce mats, baskets, hand fans, hats, cages, hot pots, salt pots, brooms, etc. in the sudy area. Hot pots, salt pots, mats, baskets, and ropes represent highly used items. The mats are used for various purposes like drying grains, performing prayers, sitting, and sleeping. As a whole, 39 different kinds of handcrafted products from the leaves were found. Our findings revealed also that other parts of the plant, ie. petioles, fruits, and bark, have been used, although more rarely, by the locals. The palm uses differ accordingly to the different cultural areas of Pakisitan, thus demonstrating that local cultural heritage significantly informs Traditional Knowledge and practices related to the use of Mazri palm. The findings suggest also that this plant represents a crucial resource for the livelihood of the local communities in dry areas of the western borders of Pakistan, starting right from the coastal areas of Baluchistan up to District Bajaur in the North, where other farming activities there are difficult due to drought conditions.

**Conclusions:**

Traditional Knowledge about the sustainable utilization of Mazri palm is eroded in Pakistan among the younger generations due to rapid globalization and industrialization processes and appropriate strategies for revitalizing this heritage in a sustainable way should be urgently fostered.

## Introduction

Plants and plant materials have always been components of the indigenous cultures throughout the world since ancient times. Fibers from plants have been of primary importance in almost all human cultures and history of processing plant fibers is more than 10,000 years old [1]. Uses of fibers in handicrafts, utensils, and other goods have a significant contribution in the evolution of cultures and ultimately people’s comforts and quality of daily life [2]. Significant numbers of these fiber goods are linked to domestic activities such as the production and use of furniture, the preparation of food, and the production of cloths [3]. Despite these facts, ethnobotanical and/or ethnographic studies focusing specifically on handcrafted products are still scarce and even more rare those studies that try to investigate cultural variations of Traditional Knowledge linked to handicrafts. Human societies, on the other hand, have used palm species since more than 10,000 years [4]. Palm leaves exhibit a large flexibility for being used in different ways and hence they have been often harvested by many local communities around the globe [5]. Moreover, palms are culturally valuble sources of foods, medicines, and especially handcrafted products [6].

Species belonging to the Arecaceae (Palmae) family are of primary importance for many traditional societies in general and in Pakistan in particular. Sixteen genera and eighteen palm species do occur in Pakistan, out of which 14 genera and 15 species are cultivated and 2 genera and 3 species are wild [7]. *Nannorrhops ritchiana* (Griff) Aitch is one of these species native to Pakistan (and Afghanistan and Iran too). It is a gregarious and versatile shrub that can survive in intense winds, severe cold, blazing heat and scarce water and can grow in extreme environments [8].

Mazri palm grows wild in different areas of Pakistan: in Sindh and West Punjab [9], Peshawar Valley, Kohat, Indus gorge [10], Kohe safid [11], Kurram valley [12], South Waziristan [13], North Waziristan [14], Frontier Region Bannu [15], Malakand [16], Hangu [17], Dera Ismail Khan [18], Mohmand [19, 20], and Sheikh Baddin National Park [21]. It is extensively distributed in a number of regions in Baluchistan [22], Mekran, Loralai [23], Khirthar National Park [24], Gawadar [25], and Shahi Tump Baluchistan [2]. It is found in depressions of sandy soil within an elevation range of 600–1100 meters a.s.l. in the Suleiman Range [26] and it forms a very patchy vegetation called *Tal* in Pashto language.

Mazri palm plays a significant role in the livelihood of the local communities and indigenous peoples of Pakistan and a considerable portion of the population of ex-FATA (Federally Administrated Tribal Areas) and Baluchistan are involved in its cultivation and in the processing of its leaves [27]. The harvesting period of this plant usually ranges from October to February. A single compound leaf yields about 30 to 40 leaflets and five kilograms of dry leaves generally give about four kilograms of products, with a usual waste of about 20% of materials [28]. Mazri palm is one of the hardest palms used as a source of fibers for weaving various utensils and rope making [2, 29]. Historically, the leaves and stems were utilized in mats, fences, and house roofing [30]. Leaves alone are were to manufacture hand fans, baskets, brooms, trays, small prayer mats, large prayer mats, grain bins, hot pots, hats, and sandals [18]. The reddish moss-like wool of the petioles of Mazri palm was sometimes utilized as tinder, while the fruits are edible and the hard-coated seeds were utilized for producing rosaries [24]. Dried leaves, stems, and peduncles of Mazri Palm were used as domestic fuel as well. In southern Europe and southern and subtropical America, the Mazri plam is grown as an ornamental plant [31]. In summary, the most interesting economical botanical use of this species is linked to the preparation of traditional handcrafted products. These products are on the verge of extinction in Pakistan due to (1) the reduction in the natural population of this palm, (2) the loss of specific ethnobotanical knowledge, and (3) the wide diffusion of synthetic fibers in the market under triggered trends of globalization, industrialization, and communication processes. Keeping in mind the importance of Mazri plant’s multifold ecosystem services, ancient hadicrafts-based ethnobotanical knowledge of rural communities, and anthropogenic and envionmental threats, the current study was aimed to:

1. document the importance of Mazri palm in terms of both ecosystemic and cultural services;
2. identify the local specific utilizations of Mazri palm based on availability of plant material and the Traditional and Indigenous Knowledge that the communities still retain;
3. understand regional and cultural variations in the uses of this palm in Pakistan;
4. possibly promote the local cultural, economic, and environmental significance of this palm and its future small-scale, sustainable manufacturing activities based on the recorded Traditional Knowledge.

## Materials and methodology

### Study area

Pakistan overall has an area of 796,095 Km^2^ and lies between the following coordinates: 60° 55′ to 75° 30′ E (longitude) and 23° 45′ to 36° 50′ N (latitude). It hosts more than 6000 species of higher plants [32] of which 70% are uni-regional and about 30% are bi-or pluri-regional distributed across four Floristic regions i.e., Irano-Turanian (45% of species), Sino-Himalayan (10%), Saharo-Sindian (9.5%), and Indian region (6%) [33]. Pakistan is custodian of four seasons, i.e., winter (December to March), spring (April to June), summer (July to September), and autumn (October to November) [34]. Most parts of the country are arid and semi-arid with the exceptions of the the southern slopes of the Himalayas and Hindu Kush, i.e., the whole of Sindh province, a major part of Baluchistan, southern parts of Punjab, and central parts of the Gilgit-Baltistan [35]. Pakistan has a rich cultural/ethnic diversity, with major ethnic groups of Pashtuns (also named Pathans or Pakhtuns), Punjabis, Baluchis, Sindhis, Gujjars, Kashmiris, Hindkowans, Chitralis, Gilgitis, Baltis, etc. who have their own languages, unique traditions, and customs.

**Fig. 1:**
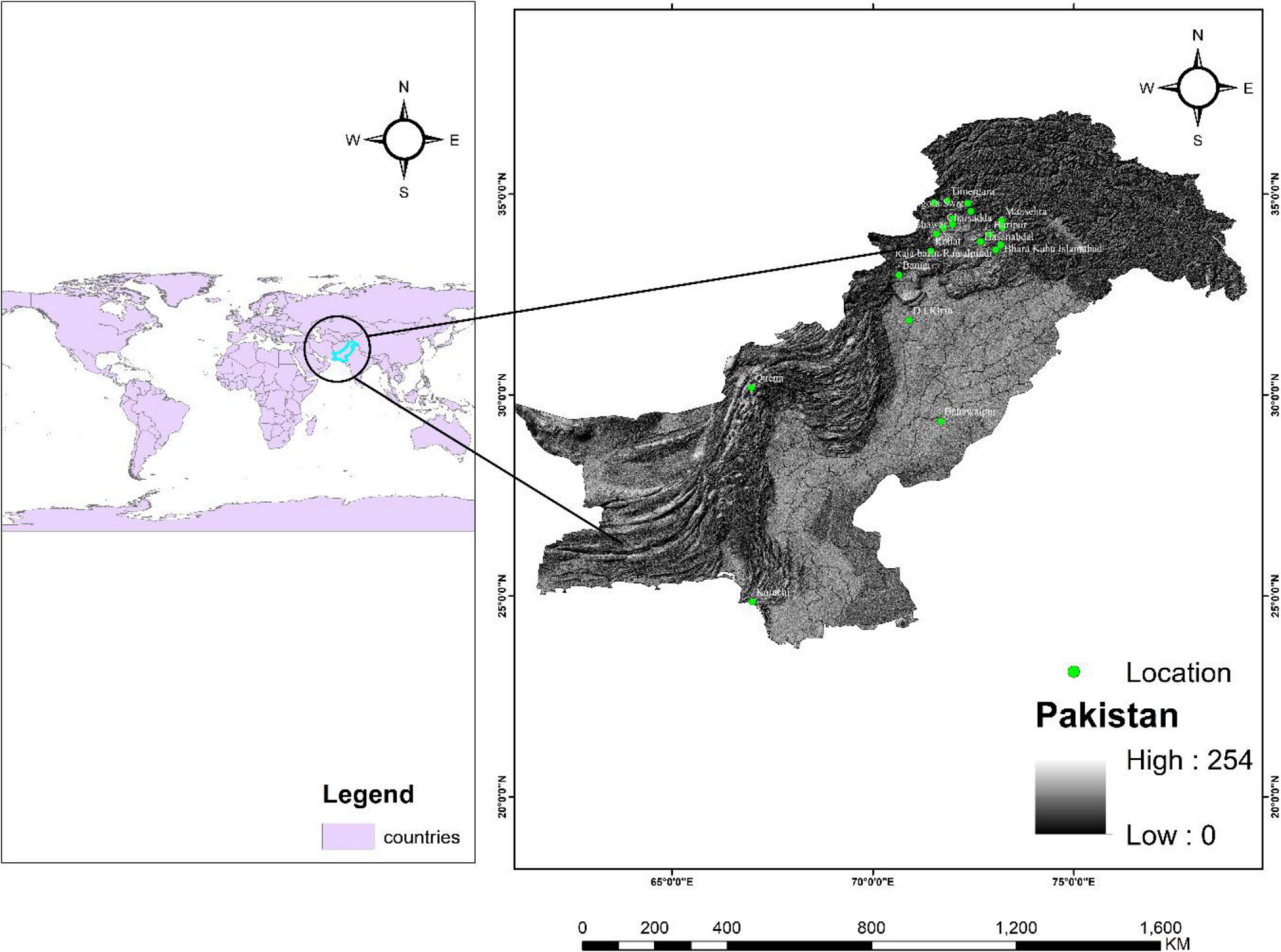
Map of the study area in Pakistan showing (in green) the localities where the surveys were conducted

We focused on diverse Mazri palm growing and Mazri manufacturing and marketing regions (Districts) of the country (Fig. 1). The Mazri growing and manufacturing areas are: Jhandai Mardan (inhabited by the following ethnic/tribal groups: Utmankhel, Mohmand, Yusafzai, and Mashwani), Lundkhwar Mardan (Utmankhel, Mohmand, Yusafzai, Gujjar), Bajaur (Tarkani, Utmankhel, Mughal, Khalji), Kohat (Bangash, Khattak, Turi, Afridi, Orakzai), Dera Ismail Khan (Kundi, Gandapur, Hasan Khel, Wazir, Mehsood, Dawar), Bannu (Banusi, Wazir, Mehsood), and Quetta (mainly populated by Pashtuns). The marketing regions include Timergara, Dir Lower (Diruji, Utmankhel, Mashwani, Tajak), Qissa Khwani Bazar Peshawar (Afghani, Yousafzai, Mohmand, Afridi, etc.), Charsadda (Umarzai, Turangzai, Sherpao, Barazai, Mohmand), Mingora Swat (Yusafzai, Kohistani), Pir Baba Buner (Bachagan, Gujjar, Sikhs, Hindus), Karachi (Muhajir, Pashtun, Sindhi, Baloch, Punjabi), Bharakahu Islamabad (Kyani, Raja, Awan, Abbasi), Raja Bazar Rawalpindi (Ghazni, Janjua, Sheikh, Gujjar, Gakhar), Hasan Abdal (Awan, Rajput, Syeds), Mansehra (Abbasi, Tanoli, Gujjar, Syeds, Awan), Abbottabad (Abbasi, Jadoon, Syeds, Gujjar, Tanoli), Haripur (Awan, Syeds, Dalazak, Rajput), and Bahawalpur (Jat, Arain, Rajput, Awan, Gujjar).

Mazri palm grows from the coastal region of Baluchistan up to the North of ex-Federally Administrated Tribal Areas (ex-FATA) and to western mountainous belt of the Khyber-Pakhtunkhwa (KP). Owing a geographically varied landscape and climatic diversification, western parts of Pakistan are gifted with rich natural resources and diverse cultures [36]. Mazri handicrafts represent a common source of livelihood for the people of those and adjacent areas; in these regions, the leaves are processed to various handcrafted products and the they are also sold in the nearby regions in fresh forms.

### Field study

The research work was carried out from the spring of 2017 until the autumn of 2018. Palm growers, Mazri farmers, handicrafts experts, manufacturers, middlemen, shopkeepers, and people related to palms marketing were selected as focus groups. These focus groups were classified in two main clusters based on their location. Focus Group 1 includes palm growers, local farmers, manufacturers, middlemen, and marketing people who live predominantly in the Pakistani-Afghan and Pakistani-Iran borders’ regions, while Focus Group 2 include business communities or shopkeepers who are localized in very specific markets in various urban areas of the country. These two different focus groups were visited and interviewed via 86 structured and semi-structured interviews, conducted with the help of an ad-hoc designed questionnaire (Additional file 1).

Border regions of Pakistan and Afghanistan (ex-FATA-Federally Administered Tribal Areas) were visited, and interviews were conducted with 17 palm growers (of which 12 were men and 5 women), 27 Mazri farmers (all men), 23 handicrafts experts/manufacturers (21 men and 2 women), and 19 sellers/middlemen. We used mainly Pashto and Urdu languages during the interviews. We started our field study from the Mazri palms farmers. They were briefed about the purpose of the collection of the data and photographs of the utensils, goods, and handicrafts made up of the Mazri palm (already stored on a tablet) were shown to them for helping us to locate manufacturers, middlemen, and further actors involved in the Mazri-business. We then approached the middlemen whom informed us about the manufacturers and markets where these handicrafted goods and utensils were produced and sold. Name, educational level, location, and profession of each interviewee were recorded. Questions related to the season of leaves collection, people involved in the collection, factors causing a threat to the plants, types of handicrafts, goods, utensils, prices and ways of transportation, etc. were asked and noted. Market values, cultural importance, and its manufacturing techniques were also asked. Interviews of Focus Group 1 were mainly related to the cultivation, culture, and economics of the Mazri palm. We then approached to Focus Group 2 that includes the local shopkeepers of the handicrafts and business communities related to Mazri handicrafts in the urban regions. Different markets were visited where 19 shopkeepers (all men) and Mazri handicrafts traders were interviewed. Questions related to prices, priorities, highly sold products, better season for sale, types of customers, supply, demand, and future resilience of the Mazri palm markets were asked during the interviews (Additional file 1). All the regions (districts) of Pakistan, which have been included in this study, host specific ethnic and tribal groups.

### Data analyses

Data were analyzed both qualitatively and quantitatively for having a pattern of use of Mazri palms among the various ethnic groups considered in both focus groups. Uses of different items prepared from Mazri palm in the areas from where it was collected and the regions from where its use was documented during the market survey were arranged in a table [37, 38].

**Table 1:** Age groups and literacy level of the considered sample

Age groups	Number of interviewed study participants	Number of male participants	Number of female participants	Percentage	Literacy level	Number of interviewed study participants	Percentage
14–25	5	5	0	5.8	Illiterate	37	43.0
26–35	11	11	0	12.8	Primary	29	33.7
36–45	17	17	0	19.8	Middle	11	12.8
46–55	22	19	3	25.6	Secondary	7	8.1
56–65	19	17	2	22.1	University	2	2.4
66–75+	12	10	2	13.9			

Multivariate statistical analyses on the obtained data were undertaken with the starting hypothesis that uses and preferences for each handcrafted products could change among the diverse considered ethnic and tribal groups as well as between rural and urban communities. Data sets of 39 different items recorded from the regions of both the focus groups at 20 stations (hosting identical ethnic groups) were analyzed in PCORD software for analysing the indigenous knowledge among these ethnicities via cluster analysis. Availability and non-availability of a specific use (1, 0) data were used for cluster and two-way cluster analyses.

The ethnoecological data documented during questionnaire surveys was quantitatively analyzed via relative frequency citation (RFC) index to show the local importance of each palm use and especially handcrafted product, following [39]:

$$ \mathrm{RFC}=\frac{\mathrm{FC}}{N}\ \left(0<\mathrm{RFC}<1\right) $$

where FC represents the number of informants mentioning a particular handcrafted product, while *N* represents the total number of the informants participated in the survey.

## Results

### Profile of the study participants

The largest proportion of informants was represented by elders, above 45 years old (61.6) (Table 1). Among the 86 informants, 43% were illiterate and 33% have had a primary education; that shows the scarce availability of formal education facilities among Traditional Knowledge holders in the study regions. Local knowledge about the items prepared from Mazri palm was common and very popular among the Focus Group 1 (tribal peoples living in close vicinity with these palms) but was decreasing rapidly among the youngsters, as well as among the Focus Group 2 members (people living far from the Mazri growing areas). There is a strong decline in marketing as well (based on interviews of Focus Group 2) due to the availability of the synthetic alternatives to Mazri handicrafts, though of very low quality and durability.

### Mazri palm processing

Preparation of fibers from Mazri leaves is a tough and laborious task. Leaves are soaked in water for 20 to 30 days until they are softened and then hammered with a wooden hammer to remove the peel. The remaining bulks of the leaves are then washed with water and rinsed into fibers and dried again. The dried fibers are then utilized for shoes, ropes, and various other handcrafted products (Table 2).

**Table 2:** Various local uses of Mazri palm in Pakistan

S. no	English name of the use or handcrafted products	Pashto name of the use or handcrafted products	Codes for two-way cluster and cluster analysis	Parts used	Relative frequency of citation	Use details
1	Hot pot (Fig. 4a)	Petwar (3 sizes)	Hot Pot	Leaves	0.76	It is used to keep breads, toasts, etc. warmer for a while after baking them
2	Salt pot (Fig. 4b)	Malge wala Lokhay	Salt-Pot	Leaves	0.65	It is used as a pot to store salt in the kitchen / kitchen table
3	Mat for beds	Kat pozakay (1 × 2 m)	Mat-Beds	Leaves	0.60	It is used as a mattress mostly during the summer season due to its insulating nature and cooling effects
4	Mat for poultry cage	Panjre da para pozakay (1 × 1 m)	Mat-P-Cg	Leaves	0.57	It is used in poultry cages to avoid grains from falling on the earth or becoming dirty
5	Mat for vehicles (Fig. 4d)	Garo wala chetai (1.5 × 1 m)	Mat-Vehi	Leaves	0.57	Conductors and drivers of heavy vehicles or trucks covering long distances use to rest on the ground or truck floor on these mats
6	Mat for grains	Dano wala Chetai (4 × 4 m)	Mat-Grai	Leaves	0.49	It is used to dry cereal grains after thrashing the crops
7	Mat for guests	Chetai melmano da para (2 × 3 m)	Mat-Gues	Leaves	0.44	It is used for setting the guest especially in large cultural gatherings and also while serving a meal
8	Prayer mat for one individual	Musala/Jai Namaz/Puzakay	P-M-O-In	Leaves	0.41	It is used for praying by a single person at home or mosque
9	Prayer mat for group of individual (Fig. 4f)	Saf/Purr (1 × 8 m)	P-M-G-In	Leaves	0.41	It is used for prayer of many people at homes as well as mosques
10	Small broom	Wara Jaro/Jarogai	S-Broom	Leaves	0.38	It is used to clean shops, rooms, vehicles, water mills, etc.
11	Large broom (Fig. 4h)	Ghata Jaro/Jaro	L-Broom	Leaves	0.38	It is used to clean large houses, mosques, office buildings, roads, and lawns
13	Hand fan (Fig. 4i)	Babozay	Hand-Fan	Leaves	0.36	They are used as hand fans during journeys and when in hot days there are breakdowns of electricity
14	Shoes for common uses (Fig. 4j)	Saflai	Sh-C-Use	Leaves	0.33	They are used in social gatherings and recreational activities
15	Shoes for ice skiing	Wawro safely	Shoe-Ice	Leaves	0.19	They were used in past to walk on ice
16	Large basket	Tokra	L-Basket	Leaves	0.21	It is used for trasporting different items from one place to another, especially food and cloths
17	Middle-size basket (Fig. 4l)	Shkarai	M-S-Bask	Leaves	0.30	It is used to keep bread warmer for longer period especially during social gatherings
18	Large-size flat basket (Fig. 4m)	Shkor/chaj	L-S-F-Bas	Leaves	0.31	It is used to remove husks from the grains by thining and shaking methods
19	Basket used in hotels	Shkor hotel wala	B-U-Hot	Leaves	0.27	It is used in hotels to keep bread warm and soft for a longer period
20	Cover for animal mouth (Fig. 4o)	Koaray da janwaro da khole da para/Bhoka	C-A-Mout	Leaves	0.22	It is used to cover the mouth of a newborn or unhealthy cattle to avoid the animal eating harmful substances. It is also used while ploughing to avoid grazing
21	Bags for packing grasses (Fig. 4p)	Kwaray	B-P-Gras	Leaves	0.24	It is used to pack fodder for cattle. It is also used by street sellers for packing steel, silver, plastic, pots, shoes, cloths, food items etc
22	Packing bags for sweets (Fig. 4q)	Pachai	P-B-Swee	Leaves	0.23	It is used for packing various kinds of baked items, rice, and unrefined sugar cane (*Gurrh*)
23	Hat (Fig. 4r)	Topay	Hat	Leaves	0.21	It is used to protect human head from heat, torrential rain, snowfall etc., during different seasons
24	Grains bin (Fig. 4s)	Tatra or Kando	Grai-Bin	Leaves	0.13	It is used to store different kinds of grains and cereals
25	Box	Petai	Box	Leaves	0.15	It is used to store varoius grains, flours and dry food items at home in farming communities
26	Ropes (Fig. 4t)	Bonr	Ropes	Leaves	0.19	It is used to produce bed steads (Fig. 4v), tying up different goods, animals, livestock, etc.
27	One seater small bed (Fig. 4u)	Katkay	O-S-S-Be	Leaves	0.28	It is used for setting a single person
28	Rope	Lange wala rasai	Rope	Leaves	0.05	It is used to form one half of the bed from where it's titghened when gets loose; that end of the bed is known as *Langa* or *Piarrma* in Pashto language
29	Rope for well	Kohi wala rasai	R-F-Wel	Leaves	0.08	Use to take water out from the wells via wheel or deeper springs
30	Ornamental plant (Fig. 4w)	Gamle wala plant	Ornament		0.02	Mazri palm is cultivated for esthetic purposes
31	-- (Fig. 4x)	Lad	Laud	Leaves	0.06	It is used to transport goods on donkeys from one place to another
32	Fuel (Fig. 4y)	Khashak/Largay	Fuel	Leaves, stem sheaths	0.09	Dry leaves, stem sheaths, and roots are used as fuel
33	Toothbrush (Fig. 4z)	Miswak	T-Brush	Petiole	0.13	Petiole of the leaf is used as a toothbrush
34	Fruit, shoot (Fig. 4aa)	Patawa	Fr-Ne-Sh	Fruits, fresh shoots	0.06	Fruits and young shoots are used as food ingredients
35	Marbles (Fig. 4ab)	Belouree	Marbles	Seeds	0.14	In some areas, kids play marble game using its hard-round seeds, and the game is locally known as *Belouree*
36	Roofs or ceiling	Sapar/Chappar	Construc	Leaves	0.03	In some areas, the leaves are used for roofing or thatching
37	Medicinal uses	Tibi istimal/Dawai	Med-Use	Leaves	0.76	Fresh leaves’ extract is used for treating stomach problems
38	Fodder	Gayah/Wakha	Animal-F	Leaves	0.65	Young leaves are grazed by animals or they are also used in powedered form
39	Cages	Panjra	B-Cages	Leaves	0.60	These are used to keep appreciated and/or birds like Chukar, Parrot, and Mayana. People also keep such bird cages in the wild in order to attract and hunt other birds (esp. in the tribal belt of Pakistan).

Different dyes such as green, blue, red, black, pink, and yellow are used to color the leaves and fibers of the Mazri palm for ornamentation purposes. Dyes are mixed with fresh boiled water, they are then continuously stirred with the help of a wooden stick till the colors are deeply absorbed into the fibers. The fibers are washed with tap water and then hanged on ropes or scattered in sand to get dry. The dried coloured leaves are then used in combination with normal leaves in various handicrafts.

### Diversity of Mazri handicrafts

Each item processed from the leaves of Mazri is valueable even though a few items only achieve more attention and maximum cash income, hence contributing to the socioeconomic uplift of the locals due to their high demand. Our findings showed that the highest preferences were recorded for hotpots followed by salt pots and mats, brooms, hand fans, shoes, and baskets. Hotpots are used in dayly life (Fig. 4a) and every house keeps hotpots for breads in order to keep them fresh and warm for a longer time after they are baked. Salt pot is used for salt packing and keeping in kitchens (Fig. 4b). Mats of different sizes are of wide use and importance for a number of purposes, e.g., prayer gatherings, drying grains, seating guests, sleep, and poultry cages and truck (Fig. 4c–f). The demand of sleeping mats increases in the summer season as these retain a cooling effect due to its insulating and hydrophilic nature. These mats do not absorb heat and water if compared to the mats made up of cotton and other synthetic materials. Brooms of different sizes are used to clean houses, shops, and other places (Fig. 4g, h) while hand fans locally known as *Babozey* are unique sort of fans used for aeration by the indigenous people where they cannot use electric fans or in the breakdowns of electricity (Fig. 4i). Traditional shoes made up of Mazri leaves are used mostly in spiritual and cultural ceremonies and gatherings (Fig. 4j). Baskets of four different types and sizes, i.e., larger, large flat, medium, and small (Fig. 4k–n), are used in the restaurants and hotels for keeping various food items warmer and safe from fermentation. Mouth cages locally known as *Bhoka* cover the mouths of livestock and are used to prevent cattle from eating harmful things or fodder during illness or while ploughing in crop fields (Fig. 4o). Bags for collecting fodder locally known as *Kwaray* used by shepherds and farmers to collect grasses and fodders, either in their fields, from the wild, or both (Fig. 4p). Bags locally known as *Pachai* are used to pick the fruits and vegetables from orchard trees and vegetable gardens (Fig. 4q). Similarly, special *Pachai* are used for packing bakery sweets and especially a sweet locally known as *Amrassae;*. Lund Khuar city in Mardan, Warrai in Dir Upper, and Batkhela in Malakand are popular cities for *Amrassae*. Hats prepared from leaves of Mazri palms are worn during prayers in the mosques as well as by labor workers to protect their heads from the sun’s heat (Fig. 4r). Grain box are used to store and keep various seed grains and flour safe from moisture, heat, and insects (Fig. 4s). Ropes for *Kats* (beds), *Khatkey* (small chairs), *Kursae* (chairs), and other purposes are prepared from the fresh and dried leaves of *Nannorrhops* locally termed as *Bonr* or *Rasai* (Fig. 4t–v). These ropes are also used to pull out water from the wells; people bind their cattles with some support via these ropes as well.

### Diversity of cultural heritage concerning Mazri palm uses

People of Pakistan in general and tribal areas in particular are well-known for their hospitality, which is strongly linked to traditional cultural norms and beliefs. *Hujra* and *Betak* (types of guests houses) are important and significant entities in this respect Local people of Baluchistan, Khyber-Pakhtunkhwa, and former Federally Administrated Tribal Areas (ex-FATA) keep different items made up of this palm in their guesthouses which are used in one way or the other as cultural obligations. They prepare certain items made up of this palm for example, *Kats*, *Khatkey*, (sofa) stools, mats for setting, mat to cover beds, mats for prayers, and hand fans which are essential part of the hospitality culture. Many people from urban regions use to visit rural areas to see experience these cultural services that promote the ecotourism sector.

**Fig. 2:**
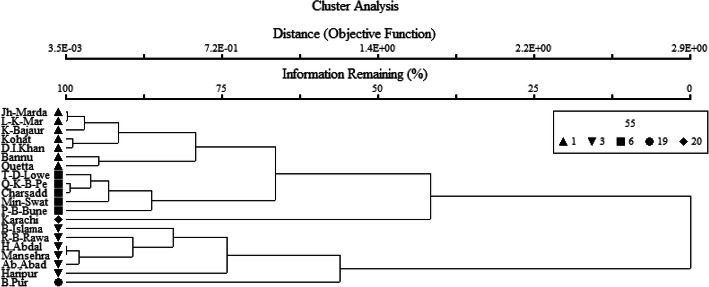
Cluster dendrogram showing the various ethnic and tribal groups who use Mazri palm in Pakistan

**Fig. 3:**
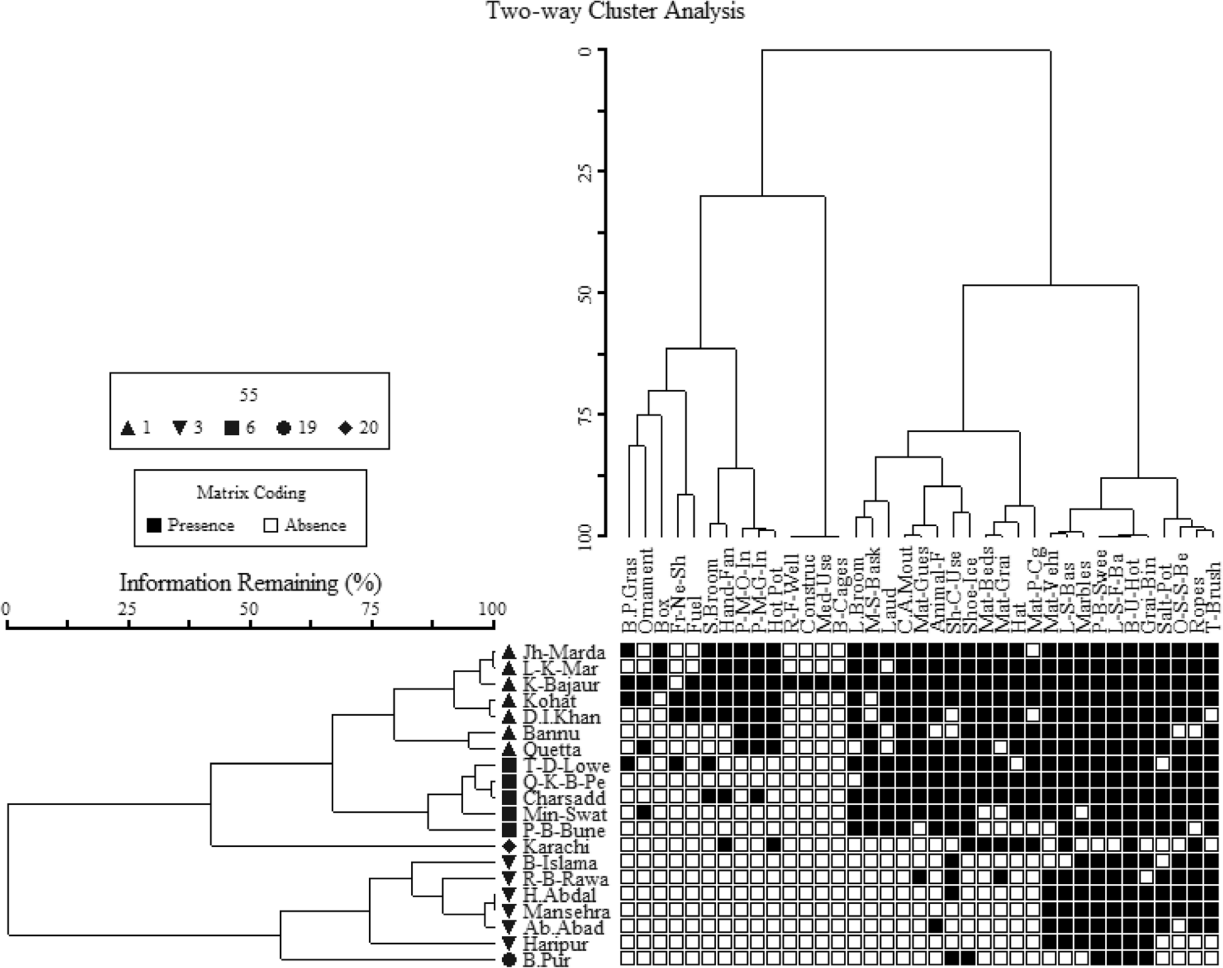
Two-way cluster dendrogram showing the distribution of the various Mazri plant uses among the different cultural zones/stations considered

Tourists visit colder areas of the country such as Swat, Muree, Abbottabad, Ayubia, Quetta, Ziarat, and others, to enjoy such hospitality cultures during the summer season. On the way to these regions, a number of markets trade such items, whose charming beauty attracts several tourists (Figs. 2 and 3).

Multivariate statistical analyses summarizing the places that have similarity and dissimilarity in uses shows that ethnoecological practices and knowledge among various ethnic groups and local communities vary from place to place, hence can be clustered into five different associations or groups (Fig. 2). A two-way cluster diagram highlights the distribution of various handicrafts among the considered ethnic groups living in different areas and with similarity in Mazri palm uses in a more comprehensive way (Fig. 3).

### Zone 01: Lower Khyber-Pakhtunkhwa and Baluchistan

The zone shown in cluster 01 comprises 7 sites, i.e., Jhandai Mardan, Lund Khwar Mardan, Khar Bajaur, Kohat, Dera Ismaiel Khan, Bannu, and Quetta, which are the areas of Lower Khyber-Pakhtunkhwa and Baluchistan where Yousafzai Pakhtuns communities live. Quetta in the province of Baluchistan is situated in the same cluster as Pakhtuns/Pathans live there as well. It shows that Pakhtuns have same cultural values and hence similar preferences for the considered kinds of goods and handicrafts irrespective of living in different provinces. Moreover, these are the areas where Mazri palms grow or have been grown in the recent past.

### Zone 02: Upper Khyber-Pakhtunkhwa

This association comprises a few localities, namely Timergara Dir Lower, Qissa Khwani Bazar Peshawar, Charsadda, Mingora Swat, and Pir Baba Buner. The people of these areas use *Nannorrhops ritchiana* for various purposes such as baskets, hand fans, brooms, and ropes. These are the colder areas of the country also known as provincially administrated tribal areas (PATA). The palm does not grow in the regions grouped in this cluster, and hence, people import it from the adjacent areas where it abundantly grows.

### Zone 03: Karachi, the only cosmopolitan city of Pakistan

The third zone is represented by Karachi the largest and the only metropolitan city of Pakistan situated on the coast of the Arabian Sea. Many peoples coming from all parts of the country use to migrate here for jobs and businesses and hence generate a multicultural environment. Peoples of Karachi use or carry different goods and utensils of Mazri and other palms from different parts of the country. That is why Karachi retains a unique position in the cluster and in the dendrograms.

### Zone 04: Hindko and Pothwari belt

Cultural zone 04 consists of Bharakahu Islamabad, Raja Bazar Rawalpindi, Hasanabdal, Haripur, Abbottabad, and Mansehra which lie in the Lesser Himalayan and Potohar plateau around the capital territory and Hazara Division. People of these areas have different culture from Pakhtuns. It is somehow a transitional between Punjabi and Pakhtun traditions. Hindku and Pahari-Pothwari are the local languages of these areas. The cluster and two-way cluster analyses separate this zone from other areas based on different kinds of uses and preference for Mazri handicrafts. This cluster also suggests a similarity in the cultures of this zone, as Hindko and Pahari-Pothwari cultures are considered as sister cultures for being custodians of closely related micro-climatic conditions/geographies, languages, and history.

### Zone 05: Bahawalpur

Bahawalpur stood out in a unique position - like Karachi - in the cluster analyses based on the recorded ethnobotanical data. This area retains a unique culture and has a peculiar history, since Bahawalpur has been the main center of the Saraiki belt, which had a long diversity of *Nawabs* (Kings) who ruled the region for hundreds of years. Bahawalpur is also special for its unique cultural heritage and palaces which were built by the its kings. The use of Mazri palm in this station includes hats, hand fans, mats, and baskets of special kind.

## Discussion

**Fig. 4:**
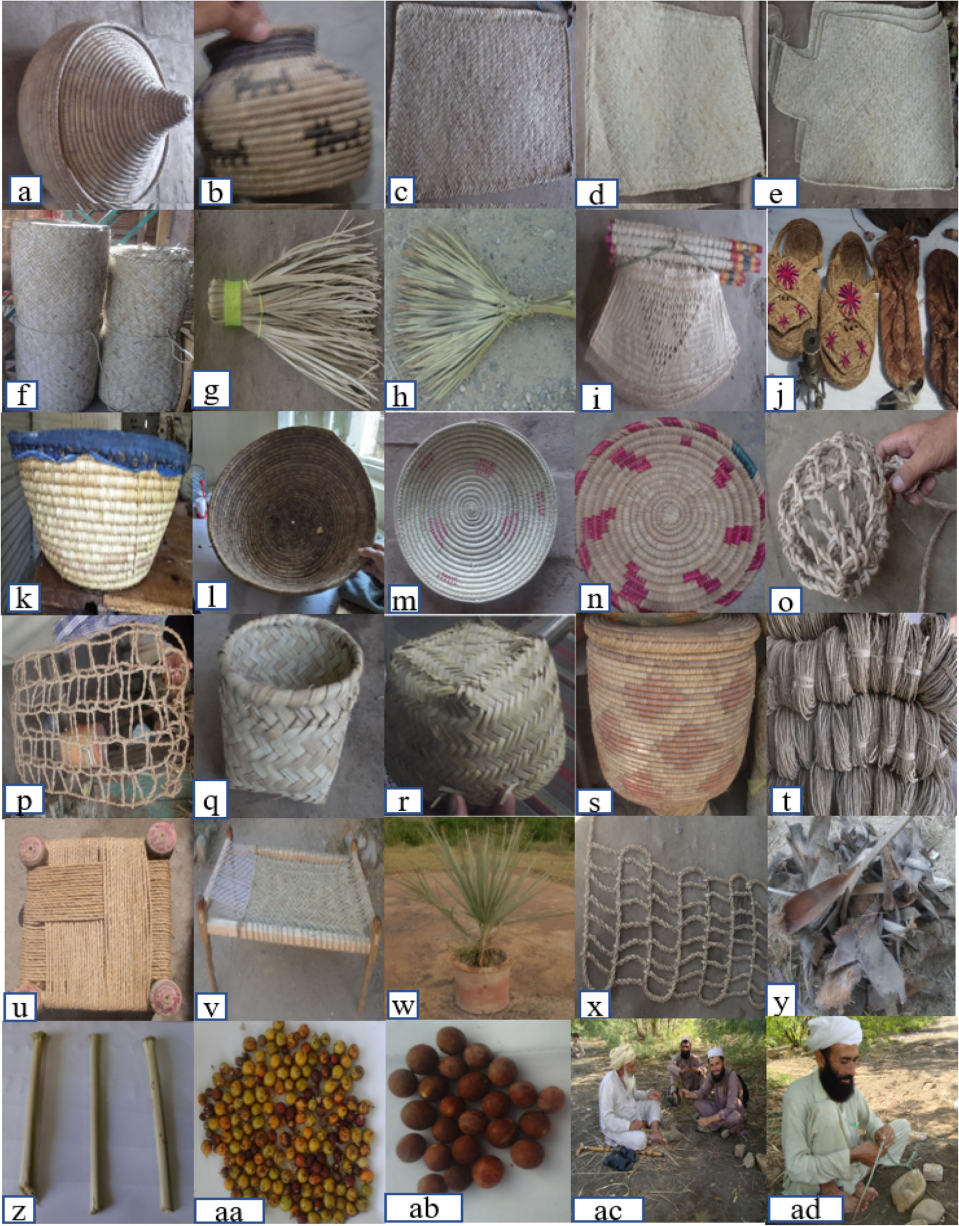
Handicrafts made up of Mazri palm. (**a**) Hot pot. (**b**) Salt pot. (**c**) Mat for beds. (**d**) Mat for vehicles. (**e**) Prayer mat for one individual. (**f**) Prayer mat for group of individuals. (**g**) Small broom. (**h**) Large broom. (**i**) Hand fan. (**j**) Shoes for common use. (**k**) Large basket. (**l**) Middle-size basket. (**m**) Large-size flat basket. (**n**) Basket used in hotels. (**o**) Cover for animal mouth. (**p**) Bags for packing grasses. (**q**) Packing bags for sweets. (**r**) Hat. (**s**) Grain bin. (**t**) Ropes. (**u**) One-seater small bed. (**v**) Cot or bed stead. (**w**) Ornamental plant. (**x**) Lad. (**y**) Fuel. (**z**) Toothbrushes. (**aa**) Fruit of Mazri palm. (**ab**) Seeds of Mazri palm used as marbles. (**ac** and **ad**) Artisans weaving ropes from Mazri leaves in Dera Ismail Khan

In the current study, we have tried to emphasize the role of Mazri palm for the local communities living in the dry climatic zones of ex-FATA and Baluchistan on the one hand and its role in the economic chain from Mazri growers to the urban business communities on the other. Local communities in the country retains different kind of Traditional Knowledge linked to this palm. The findings show that there is a considerable variation from area to area and tribe to tribe in terms of handcrafted items produced from the Mazri palm. Moreover, the data provide an important documentation of a large variety of folk uses, as fodder, food, fuel, medicine, and especially in handicrafts retained by the traditional communities. The data show that the Mazri palm has been and still is a source of fibers for weaving various utensils and ropes and for producing mats, fences, houses, roofs, hand fans, baskets, brooms, trays, prayer mats of various sizes, grain bins, hot pots, hats, and traditional sandals [2] (Fig. 4). Our findings confirm the data arising from [30] a study conducted on Baluchistain ethnobotany; these similarities may be due to the deep mutual relationship that the various ethnic communities living in Baluchistan and Khyber-Pakhtunkhwa have had across centuries, by also sharing similar cultural values and norms. According to [24], reddish moss-like wool of the petioles of *Nannorrhops* is used as tinder and the seeds are utilized for producing rosaries. In the current study, we could not record such kinds of use by locals. Findings of this article also suggest close ties between the cultural services provided by Mazri palm and human wellbeing in Pakistan and adjacent areas. High rate of exploitation of Mazri palm in the 20th Century have decreased the cultural services of this palm.

Results of cluster and two-way cluster dendrograms indicate that the people of the northwestern part of the country had more knowledge about the uses of Mazri palm if compared to those inhabiting the urban areas. Authors in other ethnobotanical studies have assessed cultural variations in a similar manner [40–42]. Another significant finding of the current study is the link between the inhabitants’ perception of various services and their resident municipalities. The maximum uses of handicrafts were found in rural areas and this can be explained by the fact that rural peoples directly collect the plant species from the wild in order to process it to handcrafted products. People from urban areas can easily reach and afford various types of artificial handicrafts and do less effort to get more durable and natural  materials from rural territories. Previously available literature on *Nannorrhops ritchiana* confirmed that this species was used for various purposes and was known by different vernacular names in various regions of cultures in Pakistan as well as in other parts of the world. These Mazri palm names vary from area to area and tribe to tribe. In Arabic language, it is called *Ghadaf* or *Sa’f* [22]; Mazri palm in English [27]; *Merez* in Afghani Pashto [43]; *Patha* in Balochistani Pashto [23]; *Mazri* in Saraiki [18]; *Purk* in Persian [44]; *Daz* in Balochi [24]; *Mezaray* in Malakand, Swat, Dir, Bajaur, and Mardan ares were Pashto is spoken [16]; *Mazara* in the Bannu Pashto [15]; and *Mazarai* in South Waziristan Pashto [13]. The palm family is one of the richest families in terms of fiber-producing plants and several researchers reported a broad variety of uses of different palm taxa in different wolrdwide regions; for example, [37] reported that the natives of Tucurui Lake in the Eastern Amazon use *Attalea speciosa* (Babassu) palm for utensils, tools, human food, animal fodder, construction, fuel, and medicines. Uses of *Attalea speciosa* reflects similarities to our findings linked to *Nannorrhops ritchiana* in the current study. Ethnoecological studies of *Braheae dulcis* showed its traditional use for two dozens of different purposes: leaves are harvested for eight decades providing several handcrafted products, mainly utilized during religious practices [45]. *Afzelia africana* is also an important palm species used for various cultural purposes in Africa. Local communities of Burkina Faso use this species as fodder, medicinal plant, food, and as raw material in carpentry. Rakotoarivelo et al. [46] studied the ethnobotanical and economic value of *Ravenala madagascariensis* in East Madagascar; according to them, the species is of immense importance for the indigenous communities and its trunk, palm heart, leaves, and petioles are the main exploited parts. Locals use them as utensils and tools, as food, and as construction material. Zambrana et al. [47] documented the context of use of *Euterpe precatoria* and *Euterpe oleracea* in Bolivia and Peru; the inhabitants of Peru use fruits and hearts of *Euterpe precatoria* palm more than the inhabitants of Bolivia for commercial and human food purposes. This findings show similarities to our data, since we recorded an important variability of Mazri palm uses among diverse cultural areas [48, 49].

*Nannorrhops ritchiana* has a great esthetic value too, can be grown in arid conditions, and hence can be recommended for being planted along main highways and motorways in Pakistan for both its beauty and for counterfighting pollution along roadsides. This opportunity could increase the production of raw material for a variety of potential handicrafts, provide some esthetic beauty to the roads, and contribute to a better environment as well. Moreover, the products prepared from the leaves of Mazri palm have major advantages if compared with artificial fibers since the palm materials are environmentally friendly, nontoxic, biodegradable, easily available, compostable, and heat resistant. These products have great capacity of absorbing sound weaves, heat, and water, while artificial fibers have instead much less virtues and cannot be easily recycled. Moreover, fibers of Mazri palm have a better elasticity and a higher toughness. Keeping the immense importance of this palm in providing crucial ecosystemic and cultural services to local populations, it is advisable that governmental and non-governmental organizations continue fostering the sustainable management of Mazri palm forests for a better future of Pakistani local communities and we sincerely hope that this paper could assist them in such endeavors.

## Conclusions

Mazri palm still play a significant role in the livelihoods of the local communities, especially in the ex-FATA belt and adjacent areas. It contributes to the livelihood of rural as well as urban populations. Handcrafted products and their utilization vary from place to place based upon diverse cultural customs and Traditional Knowledge variation is significant among the various ethnic and tribal groups. We believe that this study will offer a robust baseline of data to foster this heritage concerning the handcrafted products prepared from this palm. These findings could be also of interest for the scholars and experts working on/with plant handicrafts, especially from palm species, around the globe. We also recommend researchers and managers to bring more innovation in the further development of small-scale cottage industries by introducing new kinds of handicrafts based on Traditional Knowledge, as well as in re-envisioning community-centred and sustainable ways to organize Mazri breeding (or gathering), trade, and uses.

## Supplementary information

**Additional file 1: Appendix 1.**

## Data Availability

N/A.
