# Supplementary material for: Mazri (Nannorrhops ritchiana (Griff) Aitch.): a remarkable source of manufacturing traditional handicrafts, goods and utensils in Pakistan
Source: J Ethnobiol Ethnomed. 2020 Aug 17;16:45. doi: 10.1186/s13002-020-00394-0 (PMC7430032; doi:10.1186/s13002-020-00394-0)
Supplement: Supplementary file 1 — Additional file 1: Appendix 1. [file 13002_2020_394_MOESM1_ESM.docx]

Appendix 1: Questionnaire for the data collection related to handicrafts prepared from Mazri Palm

| **Questionnaire No. :** ……..…... **Date:** | |
| --- | --- |
| **Name:** ………………………… **Gender:** (Male / Female)  **Age:** ………………………….. **Education:** …………………………. **Profession:** …………………… **Location:** …………………………… | |
| **S. No** | **Focus Group-I: (a) Local Palm Growers/Mazri Farmers, Manufacturers, Middle men and Marketing People** |
|  | How much area you have cultivated with Mazri Palm? ................................................ |
|  | How much time Mazri palm takes to start production? ................................................. |
|  | Present Status of Mazri Palm in natural environment  a. Increasing: b. Decreasing: c. Stable: |
|  | Genders involved in Mazri Palm collection.  i) Male ii) Female |
|  | Season of Mazri Palm collection.  i) Summer ii) Winter iii) Spring iv) Fall |
|  | What is the price for 50 Kg of Mazri Palm leaves? |
|  | Who are the buyers of Mazri Palm leaves?  (i) Local People (ii) Local Contractors (iii) Direct Market Sellers. |
|  | What sort of local handicrafts/utensils they prepare from the Mazri Palm leaves? …………………………………………………………………..………………………… |
|  | Is the business of Mazri Palm being beneficial for local community? Yes/No  If yes, explain how it contributes to the socioeconomics of the locals. …………………………………………………………………………………………….. |
|  | Is it used for ornamental purposes? Yes/No |
|  | Is it used for medicinal or nutritional or other purposes? Yes/No  If yes, then what sort of use! ……………………………………………………………… |
|  | What purpose you use Mazri palm for? .............................................................................................................................................. |
|  | Do you produce handicrafts from Mazri palm? Yes/No  If yes, then what? ……………………………………….. |
|  | How many and which of the items do you currently sell in a calendar year? |
|  | What is the average price of each product?  Name of the product: …………………….. Price per item: ………………………… |
|  | Where are your primary customers?  i) Local villagers/community people (ii) Neighboring shopkeepers (iii) others ………. |
|  | Are you interested in introducing and developing your handicrafts into new/bigger markets? Yes/No |
|  | What do you need to develop your handicrafts? ............................................................................................................................................................................................................................................................................................ |

| **Focus Group-II: Business Community and Shopkeepers** | |
| --- | --- |
|  | Who are your main suppliers and from where?  ……………………………………………………………………………………………… |
|  | How many traditional handicrafts of Mazri Palm you sell? Name it also show us for photography, possibly.  ……………………………………………………………………………………………… |
|  | What kind of handicrafts has high market demand? Name please.  ……………………………………………………………………………………………… |
|  | What features do you consider important when you buy handicrafts?  …………………………………………………………………………………………….. |
|  | What kind of handicrafts has a high price? Name as well as price!  …………………………………………………………………………………………….. |
|  | Is there any specific season of the year that is best for marketing of Mazri palm’s products?  …………………………………………………………………………………………….. |
|  | Do the Mazri handicrafts are preferred by the Tourists/Visitors? |
|  | Do you have a Marketing network/chain in other cities of the country? Yes/No  If yes, where then. ………………………………………………………………………… |
|  | DO you people that variety and number of such handicrafts increase, decrease or stable with passage of time over the last 10 -15 years.  …………………………………………………………………………………………...... |
|  | Do you have any recommendation or suggestion for the development of Mazri cottage industry? ............................................................................................................................... |

The data is gathered wholly for research purposes and will be kept confidential all the time, unless and until such permissions are sought. Thanks for your time and cooperation.
